# Genetic Association of FERMT2, HLA-DRB1, CD2AP, and PTK2B Polymorphisms With Alzheimer’s Disease Risk in the Southern Chinese Population

**DOI:** 10.3389/fnagi.2020.00016

**Published:** 2020-02-04

**Authors:** Yi Yan, Aonan Zhao, Yinghui Qui, Yuanyuan Li, Ran Yan, Ying Wang, Wei Xu, Yulei Deng

**Affiliations:** ^1^Department of Neurology, Institute of Neurology, Ruijin Hospital, Shanghai Jiao Tong University School of Medicine, Shanghai, China; ^2^Department of Neurology, Ruijin Hospital, Luwan Branch, Shanghai Jiao Tong University School of Medicine, Shanghai, China

**Keywords:** Alzheimer’s disease, single nucleotide polymorphisms, FERMT2, HLA-DRB1, CD2AP, PTK2B

## Abstract

**Objectives:**

This study aimed to explore the relationship between 18 single nucleotide polymorphisms (SNPs) and Alzheimer’s disease (AD) within the southern Chinese population.

**Methods:**

A total of 420 participants, consisting of 215 AD patients and 205 sex- and age-matched controls, were recruited. The SNaPshot technique and polymer chain reaction (PCR) were used to detect the 18 SNPs. Combined with the *apolipoprotein E* (*APOE*) ε4 allele and age at onset, we performed an association analysis between these SNPs and AD susceptibility. Furthermore, we analyzed SNP-associated gene expression using the expression quantitative trait loci analysis.

**Results:**

Our study found that rs17125924 of *FERMT2* was associated with the risk of developing AD in the dominant (*P* = 0.022, odds ratio [OR] = 1.57, 95% confidence interval [CI]: 1.07–2.32) and overdominant (*P* = 0.005, OR = 1.76, 95% CI: 1.18–2.61) models. Moreover, compared with *APOE* ε4 non-carriers, the frequency of the G-allele at rs17125924 was significantly higher among AD patients in *APOE* ε4 allele carriers (*P* = 0.029). The rs9271058 of *HLA-DRB1* (dominant, overdominant, and additive models), rs9473117 of *CD2AP* (dominant and additive models), and rs73223431 of *PTK2B* (dominant, overdominant, and additive models) were associated with early onset AD (EOAD). Using the genotype-tissue expression (GTEx) and Braineac database, we found a significant association between rs9271058 genotypes and *HLA-DRB1* expression levels, while the CC genotype at rs9473117 and the TT genotype of rs73223431 increased *CD2AP* and *PTK2B* gene expression, respectively.

**Conclusion:**

Our study identifies the G-allele at rs17125924 as a risk factor for developing AD, especially in *APOE* ε4 carriers. In addition, we found that rs9271058 of *HLA-DRB1*, rs9473117 of *CD2AP*, and rs73223431 of *PTK2B* were associated with EOAD. Further studies with larger sample sizes are needed to confirm our results.

## Introduction

Alzheimer’s disease (AD), characterized by progressive memory loss and behavioral changes, accounts for two-thirds of dementia cases, posing a significant burden on the affected families and society (Fransquet et al., 2018). While familial AD reported cases are few, almost 95% of the cases are sporadic (Masters et al., 2015). The etiology of sporadic AD remains unclear, but it is likely caused by a combination of genetic and environmental risk factors (Blennow et al., 2006; Dorszewska et al., 2016). Recently, an increasing number of studies have focused on the heritability of AD, as the classical amyloid hypothesis insufficiently explains the pathogenesis of AD (Hardy, 1997; Gatz et al., 2006). The identification of AD-susceptibility loci may, therefore, provide the basis for a helpful and complementary method for the timely and reliable diagnosis of this disease (Belcavello et al., 2015). Over the past few decades, genome-wide association studies (GWAS), which overcome sample size limitations, were taken advantage of to study genetic changes that may contribute to AD. Meta-analyses of GWAS in populations of European ancestry have identified multiple susceptibility genes associated with AD, such as *CLU*, *CR1*, *MS4A4*, *CD2AP*, *CD33*, and *EPHA1* (Lambert et al., 2009; Naj et al., 2011). In East Asian studies, no significant differences were observed in the genotype and estimated allele frequency distribution of single nucleotide polymorphisms (SNPs) within *ABCA7*, *CD2AP*, and *EPHA1* (Tan et al., 2013), suggesting that the genetic basis for AD susceptibility may be ethnicity-specific. Our previous study found that rs3865444 of *CD33* and rs610932 of *MS4A6A* may contribute to AD risk in the Chinese Han population, which was in line with the findings of preceding GWAS studies (Deng et al., 2012).

Early onset AD (EOAD), with onset in individuals younger than 65 years, differs from late-onset AD (LOAD) in that-although genetic factors play an important role in both types-they are influenced by different susceptibility genes (Jiang et al., 2013; Tellechea et al., 2018). Approximately 10% of EOAD cases are familial in nature, based on autosomal dominant variants in the genes encoding amyloid precursor protein (APP), presenilin 1 (PSEN1), and presenilin 2 (PSEN2) (Kunkle et al., 2017). The remaining early onset sporadic cases are thought to be predominantly polygenic variants, the accumulation of which can result in EOAD at an early stage of life (Barber et al., 2017). The *apolipoprotein E* (*APOE*) ε4 allele, which plays a vital role in liquid absorption and redistribution, is regarded as the strongest genetic risk factor for LOAD (Liu et al., 2013; Qiu et al., 2019).

Recently, a GWAS meta-analysis of non-Hispanic whites confirmed 20 previously described LOAD risk loci and proposed 5 such novel loci including *IQCK*, *ACE*, *ADAM10*, *ADAMTS1*, and *WWOX* (Kunkle et al., 2019). In addition, 9 new susceptibility loci for AD have also been identified in a GWAS meta-analysis in individuals of European descent (Jansen et al., 2019). Repeating the GWAS results in different ethnic populations can aid in identifying the SNPs associated with AD (Chanock et al., 2007). To the best of our knowledge, due to varying allele frequencies between different races, the association of these candidate loci reported in the above GWAS with AD in the Chinese population is not known. Therefore, in this study, we selected 18 SNPs (*FERMT2* rs17125924, *HLA-DRB1* rs9271058 and rs6931277, *CD2AP* rs9473117, *APH1B* rs35408871, *NDUFAF6* rs4735340, *ADAMTS20* rs7295246, *EPHA1* rs10808026 and rs11763230, *ADAM10* rs593742 and rs442495, *INPP5D* rs10933431, *PTK2B* rs73223431, *CR1* rs2093760, *MS4A6A* rs7935829, *CLNK* rs6448451, *CD2AP-TNFRSF21* rs9381563, *CLU* rs4236673) from aforementioned studies, aiming to explore the relationship between these genes and AD risk in the southern Chinese population. Moreover, we attempted to assess the potential effect of these loci on gene expression using multiple expression quantitative trait loci (eQTL) datasets.

## Materials and Methods

### Study Population

From September 2016 to March 2019, a total of 215 patients with AD (135 women and 80 men, mean age at onset ± SD: 71.95 ± 8.46 years) were recruited from the outpatient clinic at the Department of Neurology, Ruijin Hospital, affiliated to Shanghai Jiao Tong University School of Medicine, China. All enrolled subjects were evaluated by at least two experienced neurologists and underwent a standard series of examinations, including medical history, physical examinations, as well as neuropsychological and neuroimaging tests. The results met the criteria for probable AD defined by National Institute of Neurological and Communicative Disorders and Stroke–Alzheimer’s Disease and Related Disorders Association (NINCDS-ADRDA criteria) (Dubois et al., 2007). Participants with a history of other neurological diseases that may cause dementia, such as stroke, Parkinson’s disease, brain tumor, multiple sclerosis, and major depression, were excluded (McKhann et al., 2011; Janelidze et al., 2018). The age at the onset of the disease was determined by the medical history provided by the caregiver. The control group consisted of 205 healthy volunteers matched for sex and age (121 women and 84 men, mean age ± SD: 70.74 ± 7.82 years). Healthy subjects were carefully assessed by a physician to confirm the absence of cognitive decline symptoms, thus ensuring they did not fulfill the criteria of mild cognitive impairment (MCI) or dementia (McKhann et al., 2011; Jack et al., 2018). This study was approved by the Ethics Committee of the Ruijin Hospital affiliated to the Shanghai Jiao Tong University School of Medicine (2018-No.6).

### Genotype Analysis

The genomic DNA was extracted using the phenol-chloroform-isopropyl alcohol method from 2 mL of blood collected in EDTA anti-coagulation tubes. The SNaPshot technique (Applied Biosystems, Foster City, CA, United States) was used to genotype SNPs. Polymer chain reaction (PCR) and extension primers were designed using the Primer5 software (version 5.00, PREMIER Biosoft International). The length of PCR fragments ranged from 80 to 240 bp. The PCR products were purified by phosphorylase (FastAP, Applied Biosystems) and exonuclease I (EXO I, Applied Biosystems) and subsequently extended using the ABI SNaPshot Multiplex kit (Applied Biosystems). The extension product was purified by FastAP and loaded on ABI3730xl (Applied Biosystems). The results were analyzed using GeneMapper 4.0 (Applied Biosystems). Primer sequences are listed in Supplementary Table 1. The SNPs in *APOE* (rs429358 and rs7412) were genotyped by the polymerase chain reaction–restriction fragment length polymorphism (PCR–RFLP) method as previously described (Corder et al., 1993).

### Gene Expression Analysis

Expression quantitative trait loci were examined using two different databases. The Braineac eQTL dataset is a public database developed by the United Kingdom Brain Expression Consortium (UKBEC), which includes 10 brain regions from 134 postmortem individuals of European descent without neurodegenerative disorders (Ramasamy et al., 2014). The GTEx project has collected genotypes and gene expression data from 54 non-diseased tissue sites across nearly 1,000 individuals^1^.

### Statistical Analyses

All statistical analyses were performed using the SPSS 25.0 software package (SPSS Inc., Chicago, IL, United States). Differences in age, level of education and Mini−Mental State Examination (MMSE) scores between the two groups were examined by *t*-test. Chi-square test was used to compare the differences in sex proportions and *APOE* status, as well as in allele and genotype frequencies. The Hardy-Weinberg equilibrium (HWE) of the entire cohort was also tested using the chi-square test. The risk of each SNP was estimated using logistic regression analysis after adjusting for age and sex, and four genetic models including dominant, recessive, overdominant, and additive models were applied. The following definitions were used assuming A represents the major allele and a represents the minor allele: dominant was defined as 1 (aa + Aa) versus 0 (AA); recessive as 1 (aa) versus 0 (AA + Aa); additive as 0 (AA) versus 1 (Aa) versus 2 (aa); and overdominant as 1 (Aa) versus 0 (AA + aa). *P*-values < 0.05 were considered statistically significant. Multiple tests were performed using the Bonferroni correction method. The genetic power of each SNP was calculated using Power and Sample Size software (version 3.1.6).

## Results

### The Study Population’s Demographic Characteristics

In this study, we analyzed 215 patients with AD and 205 age- and sex-matched healthy controls. Table 1 shows the main demographic and clinical information of these subjects. Compared to controls, AD patients were found to be less educated, which is consistent with the findings of previous studies (Xu et al., 2016; Larsson et al., 2017).

**TABLE 1:** Characteristics of the study population.

	Patient (*N* = 215)	Control (*N* = 205)	*P* value
Female, n (%)	135 (62.79%)	121 (59.02%)	0.429
Male, n (%)	80 (37.21%)	84 (40.98%)	–
Age at examination (years, mean ± SD)	73.67 ± 7.23	70.74 ± 7.82	0.128^a^
Age at onset	71.95 ± 8.46	–	
EOAD, n (%)	49 (22.79%)	–	
Female^b^, n (%)	34 (69.39%)	–	
Education level (years, mean ± SD)	8.35 ± 4.96	10.73 ± 3.96	**<0.001**
MMSE score (mean ± SD)	15.33 ± 5.96	28.80 ± 1.24	**<0.001**
CDT score (mean ± SD)	1.61 ± 1.47	3.94 ± 0.24	**<0.001**
APOE genotype (%)			
ε2/ε2	2 (0.93%)	1 (0.49%)	
ε2/ε3	11 (5.12%)	23 (11.22%)	
ε2/ε4	7 (3.26%)	2 (0.98%)	
ε3/ε3	89 (41.40%)	136 (66.34%)	
ε3/ε4	83 (38.60%)	42 (20.49%)	
ε4/ε4	23 (10.70%)	1 (0.49%)	
APOE ε4 carriers	113 (52.56%)	45 (21.95%)	**<0.001**

*AD, Alzheimer’s disease; Control, healthy controls; MMSE, Mini-Mental State Examination; CDT, clock drawing test; APOE, apolipoprotein E, APOE ε4 carriers have at least one copy of the ε4 allele; SD, standard deviation a: *P* value was compared with the age of onset for AD patients and age at examination for control subjects. b: Referred to EOAD. Bold indicates statistically significant values.*

### Association Analysis of SNPs With AD in Different Genetic Models

For all SNPs, genotype distributions were in HWE. The minimum allele and genotype frequencies of these SNPs are listed in Supplementary Table 2. No significant differences were found in the allele frequencies of those SNPs between AD patients and controls. In contrast, regarding SNP genotype frequencies, we found that, at rs1715924, the genotypes GG and GA conferred a higher risk for AD than the genotype AA (Table 2). The relationship between target SNP and AD risk was studied by four genetic models including dominant, recessive, overdominant, and additive model. Following adjustment for age and sex, only one SNP, rs17125924 of *FERMT2*, was associated with the risk of AD in the dominant (*P* = 0.022, odds ratio [OR] = 1.57, 95% confidence interval [CI]: 1.07–2.32) and overdominant (*P* = 0.005, OR = 1.76, 95% CI: 1.18–2.61) models (Table 3 and Supplementary Table 3). However, after Bonferroni correction, these associations did not persist. As expected, the *APOE* status increased the risk of disease (Table 1). We stratified these data by *APOE* ε4 status in order to find out whether this allele affects the relationship between SNPs and AD susceptibility. In *APOE* ε4 carriers, the allele and genotype frequencies of *FERMT2* rs17125924 were significantly different between AD patients and controls (allele: *P* = 0.029, OR = 1.895, 95% CI: 1.06–3.38; genotype: *P* = 0.036), with allele G found to be higher in the case group than in the control (Table 2). After adjustment for age and sex, the dominant, overdominant and additive models of rs17125924 were associated with AD (dominant model: *P* = 0.009, OR = 2.739, 95% CI: 1.28–5.86; overdominant model: *P* = 0.015, OR = 2.66, 95% CI: 1.21–5.85; additive model: *P* = 0.03, OR = 2.022, 95% CI: 1.07–3.82) (Table 2). These associations could not be confirmed after Bonferroni correction. In *APOE* ε4 non-carriers, there were no significant differences in the allele frequency between AD patients and controls, but we observed a lower distribution of GG homozygosity at rs17125924 in the former group, suggesting a protective effect in the recessive model (Table 2).

**TABLE 2:** Association of rs17125924 of FERMT2 with AD risk stratified by APOEε4 status.

	MAF	P allele	P genotype	Dominant	Recessive	Additive	Overdominant
		OR (95%CI)		*P* value	*P* value	*P* value	*P* value
				OR (95%CI)	OR (95%CI)	OR (95%CI)	OR (95%CI)
Total	0.293/0.249	0.149	**0.021**	**0.022**	0.353	0.141	**0.005**
		1.252		1.57	1.45	1.27	1.76
		(0.92–1.70)		(1.07–2.32)	(0.67–3.15)	(0.93–1.73)	(1.18–2.61)
ApoE ε4(+)	0.350/0.221	**0.029**	**0.036**	**0.009**	0.762	**0.03**	**0.015**
		1.895		2.74	1.24	2.02	2.66
		(1.06–3.38)		(1.28–5.86)	(0.31–4.90)	(1.07–3.82)	(1.21–5.85)
ApoE ε4(-)	0.230/0.256	0.503	**0.028**	0.757	**0.039**	0.508	0.15
		0.869		1.08	0.12	0.87	1.45
		(0.58–1.31)		(0.66–1.78)	(0.02–0.90)	(0.57–1.32)	(0.87–2.41)

*AD, Alzheimer’s disease; Allele, minor allele; MAF, minor allele frequency; OR, odds ratio; CI, confidence interval. Dominant was defined as 1 (aa + Aa) versus 0 (AA). Recessive was defined as 1 (aa) versus 0 (AA + Aa), and additive was defined as 0 (AA) versus 1 (Aa) versus 2 (aa). Overdominant was defined as 1 (Aa) versus 0 (AA + aa). (A: major allele; a: minor allele). P allele and P genotype were examined using the Chi-square test. *P* value was adjusted for gender and age with binary logistic regression. Bold indicates statistically significant values.*

**TABLE 3:** Association of SNPs of candidate genes and odds ratio to EOAD risk.

Gene	SNP	minor allele	MAF (case/control)	OR	95%CI		P allele
HLA-DRB1	rs9271058	A	0.224/0.124	2.038	1.17–3.56		**0.011**
CD2AP	rs9473117	C	0.102/0.134	0.733	0.36–1.50		0.393
PTK2B	rs73223431	T	0.337/0.249	1.533	0.95–2.47		0.077

**Gene**	**SNP**	**Dominant model (adjusted)**			**Recessive model (adjusted)**		
		**OR**	**95%CI**	***P***	**OR**	**95%CI**	***P***

HLA-DRB1	rs9271058	2.563	1.10–5.99	**0.03**	2.23	0.23–20.99	0.483
CD2AP	rs9473117	0.327	0.12–0.93	**0.035**	0.69	0.08–5.88	0.231
PTK2B	rs73223431	3.108	1.36–7.09	**0.007**	2.603	0.54–12.64	0.235

**Gene**	**SNP**	**Additive model (adjusted)**			**Overdominant model (adjusted)**		
		**OR**	**95%CI**	**P**	**OR**	**95%CI**	**P**

HLA-DRB1	rs9271058	2.23	1.07–4.68	**0.033**	2.363	1.01–5.56	**0.049**
CD2AP	rs9473117	0.39	0.16–0.93	**0.034**	0.387	0.13–1.16	0.09
PTK2B	rs73223431	2.438	1.26–4.71	**0.008**	2.523	1.11–5.72	**0.027**

*EOAD, early onset Alzheimer’s diseases; SNP, single nucleotide polymorphism; MAF, minor allele frequency; OR, odds ratio; CI, confidence interval. P allele was examined using the Chi-square test. *P* was adjusted for gender and age with binary logistic regression. Bold indicates statistically significant values.*

### Association Analysis of Subgroups Stratified by the Age of Onset

Subsequently, the AD patients were divided into two subgroups depending on whether the age of onset was below (EOAD) or above (LOAD) 65 years. Forty-nine (22.79%) of the AD patients had EOAD, 34 (69.39%) of which were women, while in LOAD patients, 101 (60.84%) were women. There were no discrepancies in sex between EOAD and LOAD patients (*P* = 0.277, chi-square test). We found that rs9271058 of *HLA-DRB1* was associated with the risk of developing EOAD in allele and genotype frequencies (allele: *P* = 0.011, OR = 2.038, 95% CI: 1.17–3.56; genotype: *P* = 0.038). Another significant discrepancy was observed in the genotype frequencies of *FERMT2* rs17125924 polymorphism (*P* = 0.024) (Supplementary Table 4). After adjustments for age and sex, rs9271058 of *HLA-DRB1* and rs73223431 of *PTK2B* were associated with the risk of EOAD in the dominant, overdominant, and additive models (rs9271058: dominant model: *P* = 0.03, OR = 2.563, 95% CI: 1.10–5.99; overdominant model: *P* = 0.049, OR = 2.363, 95% CI: 1.01–5.56; additive model: *P* = 0.033, OR = 2.23, 95% CI: 1.07–4.68; rs73223431: dominant model: *P* = 0.007, OR = 3.108, 95% CI: 1.36–7.09; overdominant model: *P* = 0.027, OR = 2.523, 95% CI: 1.11–5.72; additive model: *P* = 0.008, OR = 2.438, 95% CI: 1.26–4.71). Additionally, rs9473117 of *CD2AP* was associated with the risk of developing EOAD in both the dominant and additive models (dominant model: *P* = 0.035, OR = 0.327, 95% CI: 0.12–0.93; additive model: *P* = 0.034, OR = 0.39, 95% CI: 0.16–0.93). The results also showed an association between rs35408871 of *APH1B* and EOAD in the recessive model (*P* = 0.047, OR = 0.36, 95% CI: 0.13–0.99). However, all statistically significant differences were eliminated after Bonferroni correction (Table 3 and Supplementary Table 5). With regard to LOAD, we only found that rs11763230 of *EPHA1* may be associated with AD susceptibility using the recessive model (*P* = 0.046, OR = 0.111, 95% CI: 0.01–0.96) (Supplementary Tables 6, 7).

### Association Analysis Between SNPs and Relevant Gene Expression in Normal Human Brain

**FIGURE 1:**
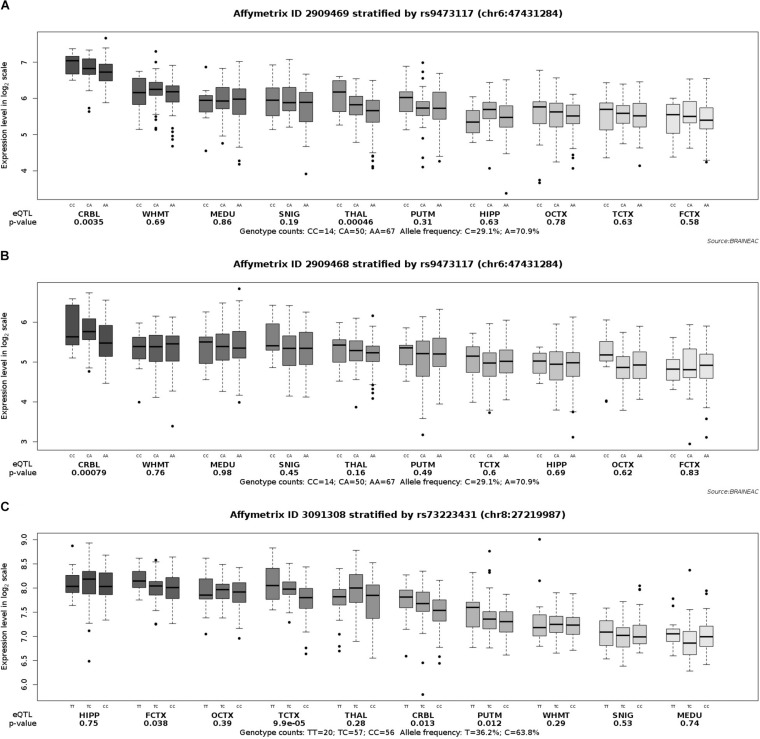
Effect of rs9473117 and rs73223431 on CD2AP and PTK2B expression in normal human brain regions in Braineac. **(A)** C allele of rs9473117 increased CD2AP expression in thalamus with probe set 2,909,469 (*P* = 4.6×10^–4^), **(B)** C allele of rs9473117 increased CD2AP expression in cerebellar cortex with probe set 2,909,468 (*P* = 7.9×10^–4^), **(C)** Carriers of T allele and especially TT genotype have more pronounced increased of PTK2B expression in temporal cortex with probe set 3,091,308 (*P* = 9.9×10^–5^).

In order to fully understand the influence of relevant loci on the onset of AD, we selected four candidate loci based on the above results and explored the association between genotype and gene expression in normal human brain by applying eQTL analysis. Using GTEx, the results show that the AA genotype of rs9271058 was associated with higher levels of *HLA-DRB1* in brain regions including the cerebellum, caudate (basal ganglia), cortex, putamen (basal ganglia), nucleus accumbens (basal ganglia), frontal cortex (BA9), cerebellar hemisphere, anterior cingulate cortex (BA24), hypothalamus, amygdala, hippocampus, and spinal cord (cervical c-1) (Table 4). Using the Braineac database, the C-allele of rs9473117 increased *CD2AP* expression in thalamus (*P* = 4.6×10^–4^, with probe set 2,909,469) and cerebellar cortex (*P* = 7.9×10^–4^ with probe set 2,909,468). In addition, the TT genotype of rs73223431 enhanced *PTK2B* expression in temporal cortex (*P* = 9.9×10^–5^, with probe set 3,091,308) (Figure 1). No significant association was found between genotypes at rs17125924 and cerebral *FERMT2* expression levels in GTEx and the Braineac database.

**TABLE 4:** Effect of rs9271058 on HLA-DRB1 gene expression in different regions of normal human brain in GTEx.

Variant ID	Tissue	Samples	Genotype			*P*-value	NES
			AA	AT	TT		
chr6_32607629_A_T_b38	Brain – Cerebellum	209	11	95	103	4.2×10^–23^	–0.72
	Brain-Caudate (basal ganglia)	194	11	80	103	1.6×10^–16^	–0.45
	Brain – Cortex	205	12	86	107	2.5×10^–15^	–0.53
	Brain – Putamen (basal ganglia)	170	11	79	80	1.3×10^–14^	–0.54
	Brain – Nucleus accumbens (basal ganglia)	202	10	83	109	4.6×10^–13^	–0.46
	Brain – Frontal Cortex (BA9)	175	7	76	92	1.3×10^–12^	–0.52
	Brain – Cerebellar Hemisphere	175	7	76	92	7.5×10^–12^	–0.57
	Brain – Anterior cingulate cortex (BA24)	147	8	67	72	1.8×10^–11^	–0.54
	Brain – Hypothalamus	170	8	69	93	1.2×10^–8^	–0.34
	Brain – Amygdala	129	5	50	74	8.9×10^–8^	–0.33
	Brain – Hippocampus	165	8	66	91	1×10^–6^	–0.27
	Brain – Spinal cord (cervical c-1)	126	7	51	68	1.4×10^–5^	–0.30

*Samples: The number of RNA-seq samples with genotype, *P*-value: from a *t*-test that compares observed NES from single-tissue eQTL analysis to a null NES of 0, NES: Normalized Effect Size, the slope of the linear regression of normalized expression data versus the three genotype categories using single-tissue eQTL analysis representing eQTL effect size.*

## Discussion

In this study, we validated an association between the rs17125924 of *FERMT2* and the risk of AD in the southern Chinese population. When the allele and genotype distribution were stratified by *APOE* ε4 status, the discrepancy was even more significant in *APOE* ε4 carriers. Individuals with a homozygous GG-allele at rs17125924 were found to have a higher susceptibility to AD. In addition, when the patients were divided into two groups according to the age at the onset of AD (EOAD and LOAD), we observed that rs9271058 of *HLA-DRB1* (dominant, overdominant, and additive models), rs9473117 of *CD2AP* (dominant and additive models), and rs73223431 of *PTK2B* (dominant, overdominant, and additive models) were possibly associated with EOAD. There was a tendency of rs35408871 within *APH1B* to be linked to the risk of EOAD in the recessive model. However, rs11763230 of *EPHA1* was the sole loci amongst our chosen SNPs that may contribute to a risk of LOAD in the recessive model. The association of the remaining 12 SNPs with AD did not replicate in the southern Chinese population.

FERMT2, also known as kindlin-2, is important in integrin activation and cell-cell adhesion (Lai-Cheong et al., 2010). As previously reported, *FERMT2* silencing leads to an increase in Aβ production, thus demonstrating its regulatory impact on APP metabolism (Chapuis et al., 2017). A new study found that *FERMT2* expression in human neurons has effects on both Aβ and Tau (Sullivan et al., 2019). Knockdown of *FERMT2* in these cells by either viral-mediated delivery of shRNA or genome editing resulted in a reduction in the extracellular Aβ40 and Aβ42 levels and a reduction in total and phospho-tau (Sullivan et al., 2019). A meta-analysis of GWAS in individuals of European ancestry identified rs17125944 of *FERMT2* as a risk factor for LOAD, even though this was inconsistent in different ethnic groups (Lambert et al., 2013). Zhang et al. (2016) could not corroborate this association in the northern Han Chinese population. No studies have yet been conducted to assess the relationship between SNP rs17125924 within the *FERMT2* gene and AD susceptibility in the Asian population. We provide evidence that the G-allele at rs17125924 located within the intronic regions of *FERMT2* is a risk factor for AD and is particularly prominent in *APOE* ε4 carriers.

Our findings suggest that rs9271058, which is located at 17793 base pairs upstream of the transcription start point of the *HLA-DRB1* gene, is associated with the risk of developing EOAD. HLA-DRB1 is a member of major histocompatibility complex Class II (MHCII), which is associated with autoimmune and infectious diseases and is a key player in the regulation of numerous immune responses (Price et al., 1999; Trowsdale and Knight, 2013). Studies have shown that immune activation and inflammation exacerbate the process of neurodegeneration in the brains of AD patients (Serpente et al., 2014; Shadfar et al., 2015). According to a GWAS-based meta-analysis, rs9271192 of *HLA-DRB1* was identified as a novel susceptibility locus associated with AD in Caucasians (Lambert et al., 2013). Furthermore, Tan et al. found that the C-allele at rs9271192 may contribute to LOAD risk in the northern Chinese population (Lu et al., 2017). The present study provides supporting evidence for the association between rs9271058 and EOAD, which should be further validated in future studies.

To date, it remains unknown whether rs9473117, which is located at 14198 base pairs downstream of the transcription start point of *CD2AP*, is related to the risk of AD in the Chinese population. CD2-associated protein (*CD2AP*) is an adaptor protein that is expressed in brain capillaries (Li et al., 2000) and binds to cortactin, a protein involved in the regulation of receptor-mediated endocytosis (Lynch et al., 2003). Case-control studies of the southern Chinese population found the C-allele at rs9296559 of *CD2AP* to be associated with the risk of sporadic AD and suggested decreased expression of *CD2AP* in peripheral blood lymphocytes of AD patients as a potential biomarker (Tao et al., 2017). In a meta-analysis of East Asian, American, Canadian, and European populations, the polymorphism of rs9349407 within *CD2AP* was shown to contribute to AD susceptibility (Chen et al., 2015). Our results demonstrated that rs9473117 near *CD2AP* is likely to be associated with EOAD, indicating that this locus may affect the onset of the disease. However, as the EOAD population included in our study was small, these observations require confirmation with larger sample sizes.

The *PTK2B* gene, which is highly expressed in the central nervous system, encodes a cytoplasmic protein tyrosine kinase (Lev et al., 1995). The interaction between *PTK2B* and hyperphosphorylated and oligomeric Tau in the brain is involved in pathophysiological processes of AD (Dourlen et al., 2017). In a mouse model of AD, the levels of *PTK2B* Tyr-402 phosphorylation were shown to be reduced in the hippocampus, and the behavioral and molecular phenotype could be rescued by *PTK2B* overexpression (Giralt et al., 2018). Although the rs28834970 of *PTK2B* has been identified to be a genetic contributor to the susceptibility of LOAD in several studies (Lambert et al., 2013; Jiao et al., 2015), no studies, to date, have investigated whether this genetic polymorphism is associated with EOAD. Thus, our study is the first to suggest a link between a *PTK2B* SNP and the risk of developing EOAD, which is demonstrated by the T-allele at rs73223431.

Both rs35408871 of *APH1B* and rs11763230 of *EPHA1* are intronic. The anterior pharynx-defective 1B (*APH1B*) is one of the four core subunits of the γ-secretase complex (Yonemura et al., 2016). *EPHA1*, located on chromosome 7q34.1, is a member of the ephrin family of tyrosine kinase receptors that play a role in cell morphology and motility, as well as in synaptic plasticity (Martinez et al., 2005). In addition, rs11767557 and rs11771145 of *EPHA1* have been associated with reduced LOAD risk (Lambert et al., 2013; Karch and Goate, 2015). In our study, we found that homozygosity for the G-allele at rs35408871 may have a protective effect on EOAD susceptibility, whereas homozygosity for the T-allele at rs11763230 may protect from LOAD susceptibility.

A GWAS meta-analysis has identified the polymorphisms rs593742 and rs442495 within *ADAM10* as novel risk loci associated with AD (Jansen et al., 2019; Kunkle et al., 2019). Our examination failed to replicate the association between these genetic loci and the risk for AD in the Chinese population, which may be explained by the heterogeneity of ethnic origin and/or small sample size. Also, we aimed to investigate the association of candidate loci with the age at the onset of AD and confirmed that the candidate genes including *HLA-DRB1*, *CD2AP*, and *PTK2B* may contribute to the development of early onset dementia. Prior studies have demonstrated that many eQTL influence the expression of local transcripts and distant genes (Battle et al., 2014). Application of eQTL database allows us to better understand human gene expression and its relationship to genetic variation, which provides a vital opportunity to explore potential functional impact for AD. Using the GTEx database, we further corroborated that the polymorphism of rs9271058 was associated with *HLA-DRB1* expression levels in several brain regions, which is consistent with the relevance between HLA-DRB1 and the pathogenesis of AD in previous GWAS results (Lambert et al., 2013; Wang et al., 2017). The differences of DNA methylation and transcriptional regulation between brain regions can significantly influence the gene expression (Kang et al., 2011). In addition, using the Braineac database, we found that the genotypes of rs9473117 and rs73223431 affected *CD2AP* and *PTK2B* gene expression, respectively. Collectively, these data prove that polymorphism at susceptible loci may affect gene expression and accelerate the onset of AD. Positive eQTL associations can illustrate the validity of this analysis to understand the roles of risk variants in disease.

This study has some limitations, the most important one being the small sample size because of its single-centered nature. In the future, we plan to perform a meta-analysis on the Asian population with an increased sample size to confirm our results. Moreover, we did not include all the novel genome-wide loci identified in the recent GWAS in our study (Jansen et al., 2019; Kunkle et al., 2019), such as *IQCK*, *ACE*, and *WWOX*, because the minor allele frequencies of these SNPs were rare. In follow-up studies, more candidate loci for AD susceptibility should be assessed. Meanwhile, regular follow up with the patients included in this study will be conducted to observe the cognitive changes and to explore the relationship between genetic polymorphisms and clinical progression.

## Conclusion

In conclusion, the SNP rs1715924 of *FERMT2* was associated with AD in the southern Chinese population, with a particularly significant risk in *APOE* ε4 carriers. This suggests that this polymorphism may interact with *APOE* to increase the AD risk in a Han Chinese population. The SNPs rs9271058 of *HLA-DRB1*, rs9473117 of *CD2AP*, and rs73223431 of *PTK2B* were found to be associated with EOAD. Considering the association between these loci and AD risk as demonstrated in here, investigating the roles of risk genes in AD pathogenesis is critical in future studies.

## Data Availability Statement

The datasets analyzed in this study can be found in the NCBI dbSNP Short Genetic Variation Database: https://www.ncbi.nlm.nih.gov/SNP/snp_viewBatch.cgi?sbid=106 3084.

## Ethics Statement

The studies involving human participants were reviewed and approved by the Ethics Committee of the Ruijin Hospital affiliated to the Shanghai Jiao Tong University School of Medicine. The patients/participants provided their written informed consent to participate in this study.

## Author Contributions

YY collected AD and control data, performed the statistical analysis, and drafted the manuscript. AZ, YQ, and RY helped to collect the AD data. YL, WX, and YW helped to collect the control data. YD designed the study and revised the manuscript.

## Conflict of Interest

The authors declare that the research was conducted in the absence of any commercial or financial relationships that could be construed as a potential conflict of interest.

## Footnotes

1 https://www.gtexportal.org/home/
