# Supplementary material for: Genetic Association of FERMT2, HLA-DRB1, CD2AP, and PTK2B Polymorphisms With Alzheimer’s Disease Risk in the Southern Chinese Population
Source: Front Aging Neurosci. 2020 Feb 4;12:16. doi: 10.3389/fnagi.2020.00016 (PMC7010721; doi:10.3389/fnagi.2020.00016)
Supplement: Supplementary file 1 [file Table_1.DOCX]

**Supplementary Table 1: Primers of tested single nucleotide polymorphisms.**

| SNPs | Forward Primers | Reverse Primers | Elongation Primers |
| --- | --- | --- | --- |
| rs17125924 | CATTCACTAATTTGCCCTTCAGCAG | ATAAAGCCAGCACCAAAGAAAGA | GCAGAGTATCACTTCAAAACTTCCT |
| rs9271058 | TTTGCAGAAGTAAGWCTTGAAGCAG | TCACTCCAGTCAATCTGGGCTT | CCAGTACTGCTCTCACTCATGC |
| rs9473117 | ACATTGCCCTGGTCGATTTCC | CCTGCTTCTTGGTCATTTTATGC | CGTGGTGACAGAAGTTAGAGAA |
| rs35408871 | ACCATTCATAACTTCCATTCATAGTGG | TGGGTTACAGCCTGTCCTTTGT | GCTAATTACAATAAAGCATGATCAAT |
| rs4735340 | TTAGGGCTGTGGGAAAGTCATC | AGGGTCCTACCGCCAGGGT | AGGCTAACTAAGGCCACAGAA |
| rs7295246 | TACTACGTGACAAGCAATCACCTAAGA | CCATCTGAATGTTCCAACCAATCTAT | GAGAGAAGTTCCTTCTAAGGGAATT |
| rs10808026 | CAGGGTCTCGTTCTGTCTGCA | TGCTTGTTCTTACCCTCCAAATC | ACCTGTAGTCCCAGCTACTTGG |
| rs593742 | AATACTAGAGGAGAATGCTTGTTGCAC | CCCAGGCTAAAATGATTCCAGGT | CCAATGCTGGTAAACATTTCAAC |
| rs10933431 | CTTAACAACTCTTTTCAGGAAGG | CACCAGCCACACTTCTGACCAA | GTGGAATGGGGAACGAAGT |
| rs73223431 | AAGGGCTAAGGGTTTCTAAAGTCGT | GTTGGGAAGGAGGATGAGTAGTGTC | GATTTACTGTTGGGATGAATTTCT |
| rs2093760 | CCCTCCAGACTCATAGTGCACAC | AGGATCAATCAGAGGAAAGCACA | AAATGGGAAGAGAGGTTCTCAA |
| rs7935829 | CCCCAGATGCCCAAACCAG | TCACTTTATACCACGGACTGCTATACA | GAGAGATTTTCAAAAGAAGGAATAAC |
| rs11763230 | CAAAATCAACTTTACTGAGGGACAACT | CTAAGGCTGGAGGTGGGATTG | TGCAGCCATTCCAAGTGTA |
| rs6448451 | AAGCGTCCTTACTGAATGCGG | GGAAAACAGGAGGGAAACAAGC | CCACCATACCATCACTCTTTTTG |
| rs9381563 | AGAAAAGGCTTTTCAACTTTAATGAA | ACACCCTGCCTGATATAGTCTGTCAA | GTTTTTATGTCATTTAGAAAGTTTAAAC |
| rs442495 | ACCTGTTTTTACAACACTGCTATGC | GTGAATTATAACCAAAGGCAGATGA | GCTTTCASACAACAGCAGAG |
| rs4236673 | GACTCTATCTCAAAGGAATCCCCTG | TAAGCATCTGGGTTGGTTCTGAT | TCCCCTGACAAAGAAAAAGAT |
| rs6931277 | ACACTGTGGGATGAGGAGATGAA | TTCTGCCTTCTATTTTTCTGGTGT | GCTTGTTTGTTTATGTGATTAACAA |

SNP: single nucleotide polymorphism

**Supplementary Table 2:** **The alleles and genotype distribution of candidate genes in the controls and the AD cases.**

| SNP | Alleles [n (%)] | | P value | Genotype [n (%)] | | | P value | HWE P value | Power |
| --- | --- | --- | --- | --- | --- | --- | --- | --- | --- |
|  |  |  | OR (95%CI) |  |  |  |  |  |  |
| rs17125924 | A | G | 0.149 | AA | AG | GG |  |  |  |
| AD | 304(0.707) | 126(0.293) | 1.252 | 101(0.470) | 102(0.474) | 12(0.056) | **0.021** | 0.105 | 0.175 |
| CON | 308(0.751) | 102(0.249) | (0.92-1.70) | 119(0.580) | 70(0.341) | 16(0.078) |  |  |  |
| rs9271058 | T | A | 0.261 | TT | AT | AA |  |  |  |
| AD | 365(0.849) | 65(0.151) | 0.798 | 155(0.721) | 55(0.256) | 5(0.023) | 0.504 | 0.487 | 0.113 |
| CON | 359(0.876) | 51(0.124) | (0.54-1.18) | 158(0.771) | 43(0.21) | 4(0.02) |  |  |  |
| rs9473117 | A | C | 0.481 | AA | AC | CC |  |  |  |
| AD | 365(0.849) | 65(0.151) | 1.149 | 158(0.735) | 49(0.228) | 8(0.037) | 0.798 | 0.504 | 0.078 |
| CON | 355(0.866) | 55(0.134) | (0.78-1.69) | 156(0.761) | 43(0.210) | 6(0.029) |  |  |  |
| rs35408871 | A | G | 0.621 | AA | AG | GG |  |  |  |
| AD | 214(0.498) | 209(0.502) | 1.071 | 53(0.247) | 108(0.502) | 54(0.251) | 0.84 | 0.889 | 0.064 |
| CON | 209(0.515) | 197(0.485) | (0.82-1.41) | 52(0.256) | 105(0.517) | 46(0.227) |  |  |  |
| rs4735340 | T | A | 0.552 | TT | AT | AA |  |  |  |
| AD | 298(0.693) | 132(0.307) | 0.914 | 98(0.456) | 102(0.474) | 15(0.070) | 0.743 | 0.389 | 0.070 |
| CON | 289(0.712) | 117(0.288) | (0.68-1.23) | 100(0.493) | 89(0.438) | 14(0.069) |  |  |  |
| rs7295246 | T | G | 0.971 | TT | TG | GG |  |  |  |
| AD | 363(0.844) | 67(0.156) | 1.007 | 151(0.702) | 61(0.284) | 3(0.014) | 0.082 | 0.723 | 0.050 |
| CON | 339(0.843) | 63(0.157) | (0.69-1.46) | 147(0.731) | 45(0.224) | 9(0.045) |  |  |  |
| rs10808026 | C | A | 0.869 | CC | CA | AA |  |  |  |
| AD | 364(0.847) | 66(0.153) | 1.032 | 151(0.702) | 62(0.288) | 2(0.009) | 0.149 | 0.473 | 0.051 |
| CON | 342(0.842) | 64(0.158) | (0.71-1.50) | 146(0.719) | 50(0.246) | 7(0.034) |  |  |  |
| rs593742 | G | A | 0.918 | GG | AG | AA |  |  |  |
| AD | 340(0.791) | 90(0.209) | 1.018 | 136(0.633) | 68(0.316) | 11(0.051) | 0.871 | 0.907 | 0.051 |
| CON | 323(0.788) | 87(0.212) | (0.73-1.42) | 127(0.620) | 69(0.337) | 9(0.044) |  |  |  |
| rs10933431 | C | G | 0.396 | CC | CG | GG |  |  |  |
| AD | 295(0.686) | 135(0.314) | 0.883 | 98(0.456) | 99(0.460) | 18(0.084) | 0.346 | 0.524 | 0.092 |
| CON | 270(0.659) | 140(0.341) | (0.66-1.18) | 91(0.444) | 88(0.429) | 26(0.127) |  |  |  |
| rs73223431 | C | T | 0.051 | CC | CT | TT |  |  |  |
| AD | 297(0.691) | 133(0.309) | 1.352 | 104(0.484) | 89(0.414) | 22(0.102) | 0.136 | 0.137 | 0.28 |
| CON | 308(0.751) | 102(0.249) | 1-1.83 | 119(0.580) | 70(0.341) | 16(0.078) |  |  |  |
| rs2093760 | G | A | 0.753 | GG | AG | AA |  |  |  |
| AD | 265(0.616) | 165(0.384) | 0.956 | 86(0.400) | 93(0.433) | 36(0.167) | 0.9 | 0.669 | 0.056 |
| CON | 257(0.627) | 153(0.373) | (0.72-1.26) | 83(0.405) | 91(0.444) | 31(0.151) |  |  |  |
| rs7935829 | A | G | 0.496 | AA | AG | GG |  |  |  |
| AD | 359(0.835) | 71(0.165) | 0.883 | 153(0.712) | 53(0.247) | 9(0.042) | 0.483 | 0.459 | 0.077 |
| CON | 335(0.817) | 75(0.183) | (0.62-1.26) | 137(0.668) | 61(0.298) | 7(0.034) |  |  |  |
| rs11763230 | C | T | 0.624 | CC | CT | TT |  |  |  |
| AD | 363(0.844) | 67(0.156) | 0.912 | 150(0.698) | 63(0.293) | 2(0.009) | 0.114 | 0.354 | 0.064 |
| CON | 341(0.832) | 69(0.168) | (0.63-1.32) | 144(0.702) | 53(0.259) | 8(0.039) |  |  |  |
| rs6448451 | C | G | 0.681 | CC | CG | GG |  |  |  |
| AD | 338(0.786) | 92(0.214) | 1.072 | 133(0.619) | 72(0.335) | 10(0.047) | 0.812 | 0.916 | 0.059 |
| CON | 327(0.798) | 83(0.202) | (0.77-1.50) | 129(0.629) | 69(0.337) | 7(0.034) |  |  |  |
| rs9381563 | T | C | 0.32 | TT | CT | CC |  |  |  |
| AD | 341(0.793) | 89(0.207) | 0.84 | 135(0.628) | 71(0.330) | 9(0.042) | 0.598 | 0.581 | 0.102 |
| CON | 333(0.820) | 73(0.180) | (0.59-1.18) | 137(0.675) | 59(0.291) | 7(0.034) |  |  |  |
| rs442495 | C | T | 0.915 | CC | CT | TT |  |  |  |
| AD | 356(0.828) | 74(0.172) | 0.981 | 147(0.684) | 62(0.288） | 6(0.028) | 0.948 | 0.961 | 0.051 |
| CON | 335(0.825) | 71(0.175) | (0.69-1.40) | 137(0.675） | 61(0.300) | 5(0.025) |  |  |  |
| rs4236673 | G | A | 0.919 | GG | AG | AA |  |  |  |
| AD | 357(0.830) | 73(0.170) | 1.019 | 148(0.688) | 61(0.284) | 6(0.028) | 0.578 | 1.000 | 0.051 |
| CON | 336(0.828) | 70(0.172) | (0.71-1.46) | 142(0.700) | 52(0.256) | 9(0.044) |  |  |  |
| rs6931277 | A | T | 0.744 | AA | AT | TT |  |  |  |
| AD | 374(0.870) | 56(0.130) | 0.936 | 162(0.753) | 50(0.233) | 3(0.014) | 0.726 | 0.921 | 0.056 |
| CON | 350(0.862) | 56(0.138) | (0.63-1.40) | 152(0.749) | 46(0.227) | 5(0.025) |  |  |  |

SNP, single nucleotide polymorphism; AD, Alzheimer's disease; CON, healthy controls; OR, odds ratio; CI, confidence interval; HWE, Hardy-Weinberg equilibrium; Power, genetic power.

Bold indicates statistically significant values.

**Supplement Table 3.** **Association of SNP of candidate genes with AD risk in four genetic models.**

| Gene | SNP | dominant model(adjusted) | | | Recessive model(adjusted) | | |
| --- | --- | --- | --- | --- | --- | --- | --- |
|  |  | OR | 95%CI | P value | OR | 95%CI | P value |
| FERMT2 | rs17125924 | 1.57 | 1.07-2.32 | **0.022** | 1.45 | 0.67-3.15 | 0.353 |
| HLA-DRB1 | rs9271058 | 1.38 | 0.88-2.16 | 0.157 | 1.22 | 0.32-4.65 | 0.772 |
| CD2AP | rs9473117 | 1.15 | 0.74-1.78 | 0.550 | 1.43 | 0.48-4.27 | 0.517 |
| APH1B | rs35408871 | 1.01 | 0.65-1.58 | 0.97 | 1.18 | 0.75-1.85 | 0.486 |
| NDUFAF6 | rs4735340 | 1.15 | 0.78-1.69 | 0.487 | 1.04 | 0.49-2.23 | 0.914 |
| ADAMTS20 | rs7295246 | 1.20 | 0.78-1.84 | 0.418 | 0.31 | 0.08-1.15 | 0.08 |
| EPHA1 | rs10808026 | 1.14 | 0.74-1.75 | 0.56 | 0.25 | 0.50-1.21 | 0.085 |
| ADAM10 | rs593742 | 0.93 | 0.63-1.39 | 0.723 | 1.12 | 0.45-2.76 | 0.812 |
| INPP5D | rs10933431 | 0.96 | 0.65-1.41 | 0.839 | 0.62 | 0.33-1.16 | 0.135 |
| PTK2B | rs73223431 | 1.45 | 0.99-2.14 | 0.059 | 1.31 | 0.67-2.59 | 0.431 |
| CR1 | rs2093760 | 1.01 | 0.69-1.50 | 0.947 | 1.11 | 0.66-1.88 | 0.693 |
| MS4A6A | rs7935829 | 0.83 | 0.54-1.25 | 0.367 | 1.27 | 0.46-3.50 | 0.64 |
| EPHA1 | rs11763230 | 1.07 | 0.70-1.63 | 0.748 | 0.22 | 0.05-1.04 | 0.057 |
| CLNK | rs6448451 | 1.01 | 0.68-1.51 | 0.948 | 1.34 | 0.50-3.61 | 0.564 |
| CD2AP-TNFRSF21 | rs9381563 | 1.23 | 0.82-1.85 | 0.312 | 1.40 | 0.50-3.89 | 0.525 |
| ADAM10 | rs442495 | 0.95 | 0.63-1.43 | 0.802 | 1.14 | 0.34-3.82 | 0.831 |
| CLU | rs4236673 | 1.06 | 0.70-1.61 | 0.792 | 0.63 | 0.22-1.81 | 0.392 |
| HLA-DRB1 | rs6931277 | 0.97 | 0.62-1.52 | 0.895 | 0.52 | 0.12-2.24 | 0.384 |
| Gene | SNP | overdominant model(adjusted) | | | additive model(adjusted) | | |
|  |  | OR | 95%CI | P value | OR | 95%CI | P value |
| FERMT2 | rs17125924 | 1.76 | 1.18-2.61 | **0.005** | 1.27 | 0.93-1.73 | 0.141 |
| HLA-DRB1 | rs9271058 | 1.38 | 0.87-2.18 | 0.174 | 1.31 | 0.88-1.95 | 0.182 |
| CD2AP | rs9473117 | 1.09 | 0.68-1.73 | 0.727 | 1.15 | 0.79-1.66 | 0.471 |
| APH1B | rs35408871 | 0.89 | 0.61-1.32 | 0.571 | 1.07 | 0.81-1.40 | 0.654 |
| NDUFAF6 | rs4735340 | 1.14 | 0.77-1.67 | 0.52 | 1.10 | 0.81-1.51 | 0.543 |
| ADAMTS20 | rs7295246 | 1.43 | 0.91-2.24 | 0.121 | 1.02 | 0.70-1.48 | 0.918 |
| EPHA1 | rs10808026 | 1.315 | 0.85-2.05 | 0.224 | 1.00 | 0.68-1.46 | 0.996 |
| ADAM10 | rs593742 | 0.91 | 0.60-1.37 | 0.636 | 0.97 | 0.69-1.34 | 0.836 |
| INPP5D | rs10933431 | 1.15 | 0.78-1.70 | 0.469 | 0.88 | 0.66-1.18 | 0.395 |
| PTK2B | rs73223431 | 1.35 | 0.91-2.01 | 0.14 | 1.31 | 0.97-1.77 | 0.075 |
| CR1 | rs2093760 | 0.96 | 0.65-1.41 | 0.822 | 1.04 | 0.79-1.36 | 0.803 |
| MS4A6A | rs7935829 | 0.78 | 0.50-1.20 | 0.254 | 0.90 | 0.63-1.28 | 0.552 |
| EPHA1 | rs11763230 | 1.26 | 0.82-1.95 | 0.297 | 0.94 | 0.65-1.36 | 0.74 |
| CLNK | rs6448451 | 0.96 | 0.64-1.45 | 0.862 | 1.05 | 0.74-1.47 | 0.797 |
| CD2AP-TNFRSF21 | rs9381563 | 1.18 | 0.78-1.79 | 0.434 | 1.21 | 0.86-1.71 | 0.282 |
| ADAM10 | rs442495 | 0.93 | 0.61-1.42 | 0.74 | 0.97 | 0.68-1.40 | 0.876 |
| CLU | rs4236673 | 1.15 | 0.75-1.78 | 0.526 | 0.99 | 0.69-1.41 | 0.943 |
| HLA-DRB1 | rs6931277 | 1.04 | 0.65-1.64 | 0.879 | 0.93 | 0.62-1.38 | 0.713 |

AD: Alzheimer’s disease，SNP: single nucleotide polymorphism, OR: odds ratio, CI: confidence interval

P value was adjusted for gender and age. Bold indicates statistically significant values.

**Supplementary Table 4: The allele and genotype distribution of candidate genes in the controls and the EOAD cases.**

| SNP | N | Alleles [n (%)] | | P value | Genotype [n (%)] | | | P value | HWE p value | power |
| --- | --- | --- | --- | --- | --- | --- | --- | --- | --- | --- |
|  |  |  |  | OR (95%CI) |  |  |  |  |  |  |
| rs17125924 |  | A | G | 0.171 | AA | AG | GG |  |  |  |
| EOAD | 49 | 67(0.684) | 31(0.316) | 1.397 | 20(0.408) | 27(0.551) | 2(0.041) | **0.024** | 0.189 | 0.173 |
| CON | 205 | 308(0.751) | 102(0.249) | (0.86-2.26) | 119(0.580) | 70(0.341) | 16(0.078) |  |  |  |
| rs9271058 |  | T | A | **0.011** | TT | AT | AA |  |  |  |
| EOAD | 49 | 76(0.776) | 22(0.224) | 2.038 | 29(0.592） | 18(0.367) | 2(0.041) | **0.038** | 0.224 | 0.442 |
| CON | 205 | 359(0.876) | 51(0.124) | (1.17-3.56) | 158(0.771) | 43(0.21) | 4(0.02) |  |  |  |
| rs9473117 |  | A | C | 0.393 | AA | AC | CC |  |  |  |
| EOAD | 49 | 88(0.898) | 10(0.102) | 0.733 | 40(0.816) | 8(0.163) | 1(0.020) | 0.706 | 0.744 | 0.075 |
| CON | 205 | 355(0.866) | 55(0.134) | (0.36-1.50) | 156(0.761) | 43(0.210) | 6(0.029) |  |  |  |
| rs35408871 |  | A | G | 0.234 | AA | AG | GG |  |  |  |
| EOAD | 49 | 57(0.582) | 41(0.418) | 0.763 | 17(0.347) | 23(0.469) | 9(0.184) | 0.427 | 0.612 | 0.132 |
| CON | 203 | 209(0.515) | 197(0.485) | (0.49-1.19) | 52(0.256) | 105(0.517) | 46(0.227) |  |  |  |
| rs4735340 |  | T | A | 0.961 | TT | AT | AA |  |  |  |
| EOAD | 49 | 70(0.714) | 28(0.286) | 0.988 | 25(0.510) | 20(0.408) | 4(0.082) | 0.906 | 1.000 | 0.050 |
| CON | 203 | 289(0.712) | 117(0.288) | (0.61-1.61) | 100(0.493) | 89(0.438) | 14(0.069) |  |  |  |
| rs7295246 |  | T | G | 0.373 | TT | TG | GG |  |  |  |
| EOAD | 49 | 79(0.806) | 19(0.194) | 1.294 | 30(0.612) | 19(0.388) | 0(0.000) | **0.029** | 0.285 | 0.110 |
| CON | 201 | 339(0.843) | 63(0.157) | (0.73-2.29) | 147(0.731) | 45(0.224) | 9(0.045) |  |  |  |
| rs10808026 |  | C | A | 0.268 | CC | CA | AA |  |  |  |
| EOAD | 49 | 78(0.796) | 20(0.204) | 1.37 | 30(0.612) | 18(0.367) | 1(0.020) | 0.219 | 0.551 | 0.139 |
| CON | 203 | 342(0.842) | 64(0.158) | 0.78-2.40 | 146(0.719) | 50(0.246) | 7(0.034) |  |  |  |
| rs593742 |  | G | A | 0.627 | GG | AG | AA |  |  |  |
| EOAD | 49 | 75(0.765) | 23(0.235) | 1.139 | 28(0.571） | 19(0.388） | 2(0.041) | 0.796 | 0.815 | 0.069 |
| CON | 205 | 323(0.788) | 87(0.212) | (0.67-1.92) | 127(0.620) | 69(0.337) | 9(0.044) |  |  |  |
| rs10933431 |  | C | G | 0.779 | CC | CG | GG |  |  |  |
| EOAD | 49 | 66(0.673) | 32(0.327) | 0.935 | 22(0.449) | 22(0.449) | 5(0.102) | 0.888 | 0.945 | 0.053 |
| CON | 205 | 270(0.659) | 140(0.341) | (0.59-1.50) | 91(0.444) | 88(0.429) | 26(0.127) |  |  |  |
| rs73223431 |  | C | T | 0.077 | CC | CT | TT |  |  |  |
| EOAD | 49 | 65(0.663) | 33(0.337) | 1.533 | 20(0.408) | 25(0.510) | 4(0.082) | 0.076 | 0.270 | 0.252 |
| CON | 205 | 308(0.751) | 102(0.249) | (0.95-2.47) | 119(0.580) | 70(0.341) | 16(0.078) |  |  |  |
| rs2093760 |  | G | A | 0.936 | GG | AG | AA |  |  |  |
| EOAD | 49 | 61(0.622) | 37(0.378) | 1.019 | 21(0.429) | 19(0.388) | 9(0.184) | 0.738 | 0.694 | 0.051 |
| CON | 205 | 257(0.627) | 153(0.373) | (0.65-1.61) | 83(0.405) | 91(0.444) | 31(0.151) |  |  |  |
| rs7935829 |  | A | G | 0.349 | AA | AG | GG |  |  |  |
| EOAD | 49 | 84(0.857) | 14(0.143) | 0.744 | 37(0.755) | 10(0.204) | 2(0.041) | 0.423 | 0.639 | 0.087 |
| CON | 205 | 335(0.817) | 75(0.183) | (0.40-1.38) | 137(0.668) | 61(0.298) | 7(0.034) |  |  |  |
| rs11763230 |  | C | T | 0.284 | CC | CT | TT |  |  |  |
| EOAD | 49 | 77(0.786) | 21(0.214) | 1.348 | 29(0.592) | 19(0.388) | 1(0.020) | 0.18 | 0.561 | 0.133 |
| CON | 205 | 341(0.832) | 69(0.168) | (0.78-2.33) | 144(0.702) | 53(0.259) | 8(0.039) |  |  |  |
| rs6448451 |  | C | G | 0.849 | CC | CG | GG |  |  |  |
| EOAD | 49 | 79(0.806) | 19(0.194) | 0.948 | 31(0.633) | 17(0.347) | 1(0.020) | 0.882 | 0.834 | 0.050 |
| CON | 205 | 327(0.798) | 83(0.202) | (0.54-1.65) | 129(0.629) | 69(0.337) | 7(0.034) |  |  |  |
| rs9381563 |  | T | C | 0.883 | TT | CT | CC |  |  |  |
| EOAD | 49 | 81(0.827) | 17(0.173) | 0.957 | 33(0.673) | 15(0.306) | 1(0.020) | 0.87 | 0.832 | 0.049 |
| CON | 203 | 333(0.820) | 73(0.180) | (0.54-1.71) | 137(0.675) | 59(0.291) | 7(0.034) |  |  |  |
| rs442495 |  | C | T | 0.838 | CC | CT | TT |  |  |  |
| EOAD | 49 | 80(0.816) | 18(0.184) | 1.062 | 32(0.653) | 16(0.327) | 1(0.020) | 0.93 | 0.786 | 0.055 |
| CON | 203 | 335(0.825) | 71(0.175) | (0.60-1.88) | 137(0.675) | 61(0.300) | 5(0.025) |  |  |  |
| rs4236673 |  | G | A | 0.333 | GG | AG | AA |  |  |  |
| EOAD | 49 | 77(0.786) | 21(0.214) | 1.309 | 31(0.633) | 15(0.306) | 3(0.061) | 0.649 | 0.556 | 0.118 |
| CON | 203 | 336(0.828) | 70(0.172) | (0.76-2.26) | 142(0.700) | 52(0.256) | 9(0.044) |  |  |  |
| rs6931277 |  | A | T | 0.699 | AA | AT | TT |  |  |  |
| EOAD | 49 | 83(0.847) | 15(0.153) | 1.13 | 34(0.694) | 15(0.306) | 0(0.000) | 0.301 | 0.499 | 0.065 |
| CON | 203 | 350(0.862) | 56(0.138) | (0.61-2.10) | 152(0.749) | 46(0.227) | 5(0.025) |  |  |  |

SNP, single nucleotide polymorphism; EOAD, early-onset Alzheimer’s disease; CON, healthy controls; OR, odds ratio, CI, confidence interval HWE, Hardy-Weinberg equilibrium; Power, genetic power; N, number.

Bold indicates statistically significant values.

**Supplementary Table 5: Association of SNPs of candidate genes with EOAD risk in four genetic model.**

| Gene | SNP | dominant model(adjusted) | | | Recessive model(adjusted) | | |
| --- | --- | --- | --- | --- | --- | --- | --- |
|  |  | OR | 95%CI | P value | OR | 95%CI | P value |
| FERMT2 | rs17125924 | 1.823 | 0.81-4.09 | 0.145 | 0.56 | 0.11-2.96 | 0.491 |
| HLA-DRB1 | rs9271058 | 2.563 | 1.10-5.99 | **0.03** | 2.23 | 0.23-20.99 | 0.483 |
| CD2AP | rs9473117 | 0.327 | 0.12-0.93 | **0.035** | 0.69 | 0.08-5.88 | 0.231 |
| APH1B | rs35408871 | 0.703 | 0.30-1.66 | 0.421 | 0.36 | 0.13-0.99 | **0.047** |
| NDUFAF6 | rs4735340 | 1.181 | 0.53-2.62 | 0.682 | 0.48 | 0.10-2.31 | 0.359 |
| ADAMTS20 | rs7295246 | 1.262 | 0.55-2.89 | 0.582 | / | / | / |
| EPHA1 | rs10808026 | 0.867 | 0.37-2.01 | 0.74 | 0.561 | 0.04-8.99 | 0.683 |
| ADAM10 | rs593742 | 1.862 | 0.81-4.29 | 0.145 | 0.879 | 0.09-8.71 | 0.912 |
| INPP5D | rs10933431 | 0.715 | 0.32-1.59 | 0.412 | 1.643 | 0.48-5.66 | 0.431 |
| PTK2B | rs73223431 | 3.108 | 1.36-7.09 | **0.007** | 2.603 | 0.54-12.64 | 0.235 |
| CR1 | rs2093760 | 1.149 | 0.51-2.59 | 0.738 | 1.74 | 0.63-4.80 | 0.285 |
| MS4A6A | rs7935829 | 0.784 | 0.33-1.89 | 0.586 | 0.79 | 0.10-6.11 | 0.821 |
| EPHA1 | rs11763230 | 0.902 | 0.39-2.07 | 0.808 | 0.503 | 0.03-7.38 | 0.616 |
| CLNK | rs6448451 | 0.996 | 0.43-2.31 | 0.993 | 0.366 | 0.02-6.27 | 0.488 |
| CD2AP-TNFRSF21 | rs9381563 | 0.666 | 0.29-1.56 | 0.349 | 0.129 | 0.01-1.54 | 0.106 |
| ADAM10 | rs442495 | 1.457 | 0.62-3.45 | 0.393 | 0.3 | 0.02-4.91 | 0.398 |
| CLU | rs4236673 | 1.395 | 0.59-3.28 | 0.445 | 0.965 | 0.14-6.60 | 0.971 |
| HLA-DRB1 | rs6931277 | 1.302 | 0.53-3.17 | 0.561 | / | / | / |
| Gene | SNP | overdominant model(adjusted) | | | additive model(adjusted) | | |
|  |  | OR | 95%CI | P value | OR | 95%CI | P value |
| FERMT2 | rs17125924 | 2.169 | 0.96-4.90 | 0.062 | 1.31 | 0.71-2.43 | 0.394 |
| HLA-DRB1 | rs9271058 | 2.363 | 1.01-5.56 | **0.049** | 2.23 | 1.07-4.68 | **0.033** |
| CD2AP | rs9473117 | 0.387 | 0.13-1.16 | 0.09 | 0.39 | 0.16-0.93 | **0.034** |
| APH1B | rs35408871 | 1.58 | 0.69-3.62 | 0.279 | 0.64 | 0.37-1.09 | 0.099 |
| NDUFAF6 | rs4735340 | 1.487 | 0.66-3.38 | 0.343 | 0.98 | 0.53-1.81 | 0.936 |
| ADAMTS20 | rs7295246 | 1.489 | 0.64-3.45 | 0.354 | 1.068 | 0.51-2.23 | 0.862 |
| EPHA1 | rs10808026 | 0.916 | 0.39-2.15 | 0.839 | 0.851 | 0.40-1.82 | 0.677 |
| ADAM10 | rs593742 | 1.96 | 0.83-4.61 | 0.124 | 1.59 | 0.76-3.31 | 0.216 |
| INPP5D | rs10933431 | 0.582 | 0.26-1.32 | 0.196 | 0.923 | 0.50-1.70 | 0.798 |
| PTK2B | rs73223431 | 2.523 | 1.11-5.72 | **0.027** | 2.438 | 1.26-4.71 | **0.008** |
| CR1 | rs2093760 | 0.807 | 0.35-1.85 | 0.613 | 1.239 | 0.72-2.13 | 0.437 |
| MS4A6A | rs7935829 | 0.803 | 0.32-2.02 | 0.64 | 0.821 | 0.40-1.70 | 0.596 |
| EPHA1 | rs11763230 | 0.969 | 0.42-2.25 | 0.942 | 0.87 | 0.41-1.83 | 0.713 |
| CLNK | rs6448451 | 1.112 | 0.47-2.63 | 0.809 | 0.919 | 0.44-1.94 | 0.824 |
| CD2AP-TNFRSF21 | rs9381563 | 0.915 | 0.38-2.19 | 0.841 | 0.601 | 0.29-1.24 | 0.167 |
| ADAM10 | rs442495 | 1.659 | 0.69-3.99 | 0.258 | 1.246 | 0.56-2.75 | 0.587 |
| CLU | rs4236673 | 1.451 | 0.60-3.54 | 0.413 | 1.243 | 0.62-2.50 | 0.542 |
| HLA-DRB1 | rs6931277 | 1.433 | 0.58-3.53 | 0.435 | 1.167 | 0.51-2.67 | 0.714 |

EOAD: early-onset Alzheimer’s Disease，SNP: single nucleotide polymorphism, OR: odds ratio, CI: confidence interval

P value was adjusted for gender and age. Bold indicates statistically significant values.

**Supplementary Table 6: The allele and genotype distribution of candidate genes in the controls and the LOAD cases.**

| SNP | N | Alleles [n (%)] | | P value | Genotype n(%) | | | P value | HWE p value | power |
| --- | --- | --- | --- | --- | --- | --- | --- | --- | --- | --- |
|  |  |  |  | OR (95%CI) |  |  |  |  |  |  |
| rs17125924 |  | A | G | 0.252 | AA | AG | GG |  |  |  |
| LOAD | 166 | 237(0.714) | 95(0.286) | 1.21 | 81(0.488) | 75(0.452） | 10(0.060） | 0.094 | 0.333 | 0.128 |
| CON | 205 | 308(0.751) | 102(0.249) | (0.87-1.68) | 119(0.580) | 70(0.341) | 16(0.078) |  |  |  |
| rs9271058 |  | T | A | 0.835 | TT | AT | AA |  |  |  |
| LOAD | 166 | 289(0.870) | 43(0.130) | 1.047 | 126(0.759) | 37(0.223) | 3(0.018) | 0.951 | 0.991 | 0.053 |
| CON | 205 | 359(0.876) | 51(0.124) | (0.68-1.62) | 158(0.771) | 43(0.21) | 4(0.02) |  |  |  |
| rs9473117 |  | A | C | 0.23 | AA | AC | CC |  |  |  |
| LOAD | 166 | 277(0.834) | 55(0.166) | 1.282 | 118(0.711) | 41(0.247) | 7(0.042) | 0.52 | 0.407 | 0.138 |
| CON | 205 | 355(0.866) | 55(0.134) | (0.85-1.92) | 156(0.761) | 43(0.210) | 6(0.029) |  |  |  |
| rs35408871 |  | A | G | 0.258 | AA | AG | GG |  |  |  |
| LOAD | 166 | 157(0.473) | 175(0.527) | 1.183 | 36(0.217) | 85(0.512) | 45(0.271) | 0.515 | 0.535 | 0.126 |
| CON | 203 | 209(0.515) | 197(0.485) | (0.89-1.58) | 52(0.256) | 105(0.517) | 46(0.227) |  |  |  |
| rs4735340 |  | T | A | 0.459 | TT | AT | AA |  |  |  |
| LOAD | 166 | 228(0.687) | 104(0.313) | 1.127 | 73(0.440) | 82(0.494) | 11(0.066) | 0.559 | 0.296 | 0.083 |
| CON | 203 | 289(0.712) | 117(0.288) | (0.82-1.55) | 100(0.493) | 89(0.438) | 14(0.069) |  |  |  |
| rs7295246 |  | T | G | 0.648 | TT | TG | GG |  |  |  |
| LOAD | 166 | 284(0.855) | 48(0.145) | 0.909 | 121(0.729) | 42(0.253) | 3(0.018) | 0.315 | 0.893 | 0.061 |
| CON | 201 | 339(0.843) | 63(0.157) | (0.61-1.37) | 147(0.731) | 45(0.224) | 9(0.045) |  |  |  |
| rs10808026 |  | C | A | 0.469 | CC | CA | AA |  |  |  |
| LOAD | 166 | 286(0.861) | 46(0.139) | 0.859 | 121(0.729) | 44(0.265) | 1(0.006) | 0.17 | 0.399 | 0.079 |
| CON | 203 | 342(0.842) | 64(0.158) | (0.57-1.30) | 146(0.719) | 50(0.246) | 7(0.034) |  |  |  |
| rs593742 |  | G | A | 0.729 | GG | AG | AA |  |  |  |
| LOAD | 166 | 265(0.798) | 67(0.202) | 0.939 | 108(0.651) | 49(0.295) | 9(0.054) | 0.659 | 0.659 | 0.056 |
| CON | 205 | 323(0.788) | 87(0.212) | (0.66-1.34) | 127(0.620) | 69(0.337) | 9(0.044) |  |  |  |
| rs10933431 |  | C | G | 0.368 | CC | CG | GG |  |  |  |
| LOAD | 166 | 229(0.690) | 103(0.310) | 0.867 | 76(0.458) | 77(0.464) | 13(0.078) | 0.31 | 0.533 | 0.097 |
| CON | 205 | 270(0.659) | 140(0.341) | (0.64-1.18) | 91(0.444) | 88(0.429) | 26(0.127) |  |  |  |
| rs73223431 |  | C | T | 0.111 | CC | CT | TT |  |  |  |
| LOAD | 166 | 232(0.699) | 100(0.301) | 1.302 | 84(0.506) | 64(0.386) | 18(0.108) | 0.309 | 0.237 | 0.205 |
| CON | 205 | 308(0.751) | 102(0.249) | (0.94-1.80) | 119(0.580) | 70(0.341) | 16(0.078) |  |  |  |
| rs2093760 |  | G | A | 0.73 | GG | AG | AA |  |  |  |
| LOAD | 166 | 204(0.614) | 128(0.386) | 1.054 | 65(0.392) | 74(0.446) | 27(0.163) | 0.943 | 0.808 | 0.057 |
| CON | 205 | 257(0.627) | 153(0.373) | (0.78-1.42) | 83(0.405) | 91(0.444) | 31(0.151) |  |  |  |
| rs7935829 |  | A | G | 0.691 | AA | AG | GG |  |  |  |
| LOAD | 166 | 275(0.828) | 57(0.172) | 0.926 | 116(0.699) | 43(0.259) | 7(0.042) | 0.681 | 0.749 | 0.058 |
| CON | 205 | 335(0.817) | 75(0.183) | (0.63-1.35) | 137(0.668) | 61(0.298) | 7(0.034) |  |  |  |
| rs11763230 |  | C | T | 0.266 | CC | CT | TT |  |  |  |
| LOAD | 166 | 286(0.861) | 46(0.139) | 0.795 | 121(0.729) | 44(0.265) | 1(0.006) | 0.121 | 0.239 | 0.121 |
| CON | 205 | 341(0.832) | 69(0.168) | (0.53-1.19) | 144(0.702) | 53(0.259) | 8(0.039) |  |  |  |
| rs6448451 |  | C | G | 0.562 | CC | CG | GG |  |  |  |
| LOAD | 166 | 259(0.780) | 73(0.220) | 1.11 | 102(0.614) | 55(0.331) | 9(0.054) | 0.639 | 0.834 | 0.070 |
| CON | 205 | 327(0.798) | 83(0.202) | (0.78-1.58) | 129(0.629) | 69(0.337) | 7(0.034) |  |  |  |
| rs9381563 |  | T | C | 0.207 | TT | CT | CC |  |  |  |
| LOAD | 166 | 260(0.783) | 72(0.217) | 1.263 | 102(0.614) | 56(0.337) | 8(0.048) | 0.455 | 0.443 | 0.147 |
| CON | 203 | 333(0.820) | 73(0.180) | (0.88-1.82) | 137(0.675) | 59(0.291) | 7(0.034) |  |  |  |
| rs442495 |  | C | T | 0.824 | CC | CT | TT |  |  |  |
| LOAD | 166 | 276(0.831) | 56(0.169) | 0.957 | 115(0.693) | 46(0.277) | 5(0.030) | 0.854 | 0.970 | 0.052 |
| CON | 203 | 335(0.825) | 71(0.175) | (0.65-1.41) | 137(0.675) | 61(0.300) | 5(0.025) |  |  |  |
| rs4236673 |  | G | A | 0.566 | GG | AG | AA |  |  |  |
| LOAD | 166 | 280(0.843) | 52(0.157) | 0.891 | 117(0.705) | 46(0.277) | 3(0.018) | 0.352 | 0.760 | 0.068 |
| CON | 203 | 336(0.828) | 70(0.172) | (0.60-1.32) | 142(0.700) | 52(0.256) | 9(0.044) |  |  |  |
| rs6931277 |  | A | T | 0.564 | AA | AT | TT |  |  |  |
| LOAD | 166 | 291(0.877) | 41(0.123) | 0.881 | 128(0.771) | 35(0.211) | 3(0.018) | 0.842 | 0.805 | 0.068 |
| CON | 203 | 350(0.862) | 56(0.138) | (0.57-1.36) | 152(0.749) | 46(0.227) | 5(0.025) |  |  |  |

SNP, single nucleotide polymorphism; LOAD, late-onset Alzheimer’s disease; CON, healthy controls; OR, odds ratio, CI, confidence interval HWE, Hardy-Weinberg equilibrium; Power, genetic power; N, number.

Bold indicates statistically significant values.

**Supplementary Table 7: Association of SNPs of candidate genes with LOAD risk in four genetic model.**

| Gene | SNP | dominant model(adjusted) | | | Recessive model(adjusted) | | |
| --- | --- | --- | --- | --- | --- | --- | --- |
|  |  | OR | 95%CI | P value | OR | 95%CI | P value |
| FERMT2 | rs17125924 | 1.45 | 0.94-2.25 | 0.096 | 0.827 | 0.35-1.98 | 0.67 |
| HLA-DRB1 | rs9271058 | 1.255 | 0.75-2.11 | 0.392 | 0.743 | 0.15-3.81 | 0.722 |
| CD2AP | rs9473117 | 1.403 | 0.85-2.31 | 0.182 | 2.283 | 0.69-7.61 | 0.179 |
| APH1B | rs35408871 | 1.19 | 0.71-2.00 | 0.512 | 1.564 | 0.94-2.61 | 0.087 |
| NDUFAF6 | rs4735340 | 1.211 | 0.78-1.88 | 0.391 | 1.051 | 0.44-2.52 | 0.911 |
| ADAMTS20 | rs7295246 | 1.109 | 0.68-1.82 | 0.681 | 0.448 | 0.11-1.77 | 0.252 |
| EPHA1 | rs10808026 | 1.078 | 0.66-1.77 | 0.766 | 0.119 | 0.01-1.04 | 0.055 |
| ADAM10 | rs593742 | 0.746 | 0.47-1.17 | 0.205 | 0.977 | 0.36-2.63 | 0.964 |
| INPP5D | rs10933431 | 0.978 | 0.63-1.51 | 0.92 | 0.498 | 0.24-1.04 | 0.064 |
| PTK2B | rs73223431 | 1.149 | 0.74-1.78 | 0.533 | 1.163 | 0.56-2.43 | 0.689 |
| CR1 | rs2093760 | 1.046 | 0.67-1.63 | 0.842 | 1.077 | 0.60-1.95 | 0.806 |
| MS4A6A | rs7935829 | 0.918 | 0.58-1.47 | 0.719 | 1.69 | 0.54-5.29 | 0.367 |
| EPHA1 | rs11763230 | 0.988 | 0.61-1.61 | 0.96 | 0.111 | 0.01-0.96 | **0.046** |
| CLNK | rs6448451 | 0.945 | 0.60-1.49 | 0.808 | 1.969 | 0.67-5.80 | 0.219 |
| CD2AP-TNFRSF21 | rs9381563 | 1.38 | 0.87-2.18 | 0.167 | 2.429 | 0.78-7.59 | 0.127 |
| ADAM10 | rs442495 | 0.832 | 0.52-1.33 | 0.44 | 1.107 | 0.29-4.22 | 0.882 |
| CLU | rs4236673 | 0.868 | 0.54-1.40 | 0.563 | 0.329 | 0.08-1.32 | 0.116 |
| HLA-DRB1 | rs6931277 | 0.879 | 0.53-1.47 | 0.623 | 0.525 | 0.11-2.43 | 0.409 |
| Gene | SNP | overdominant model(adjusted) | | | additive model(adjusted) | | |
|  |  | OR | 95%CI | P value | OR | 95%CI | P value |
| FERMT2 | rs17125924 | 1.548 | 0.99-2.42 | 0.055 | 1.229 | 0.87-1.74 | 0.246 |
| HLA-DRB1 | rs9271058 | 1.316 | 0.77-2.25 | 0.316 | 1.169 | 0.74-1.86 | 0.509 |
| CD2AP | rs9473117 | 1.242 | 0.74-2.09 | 0.414 | 1.397 | 0.92-2.11 | 0.114 |
| APH1B | rs35408871 | 0.812 | 0.52-1.26 | 0.356 | 1.269 | 0.92-1.75 | 0.143 |
| NDUFAF6 | rs4735340 | 1.195 | 0.77-1.85 | 0.423 | 1.146 | 0.80-1.64 | 0.454 |
| ADAMTS20 | rs7295246 | 1.273 | 0.76-2.13 | 0.357 | 0.989 | 0.65-1.50 | 0.959 |
| EPHA1 | rs10808026 | 1.308 | 0.79-2.18 | 0.301 | 0.921 | 0.59-1.43 | 0.713 |
| ADAM10 | rs593742 | 0.735 | 0.46-1.18 | 0.198 | 0.817 | 0.56-1.19 | 0.289 |
| INPP5D | rs10933431 | 1.271 | 0.82-1.97 | 0.286 | 0.853 | 0.61-1.19 | 0.343 |
| PTK2B | rs73223431 | 1.096 | 0.70-1.72 | 0.691 | 1.115 | 0.80-1.55 | 0.517 |
| CR1 | rs2093760 | 1.003 | 0.65-1.55 | 0.989 | 1.043 | 0.77-1.42 | 0.791 |
| MS4A6A | rs7935829 | 0.829 | 0.51-1.35 | 0.451 | 1.001 | 0.67-1.49 | 0.994 |
| EPHA1 | rs11763230 | 1.212 | 0.73-2.00 | 0.453 | 0.851 | 0.55-1.31 | 0.465 |
| CLNK | rs6448451 | 0.83 | 0.52-1.32 | 0.434 | 1.047 | 0.72-1.53 | 0.813 |
| CD2AP-TNFRSF21 | rs9381563 | 1.209 | 0.76-1.93 | 0.429 | 1.401 | 0.95-2.07 | 0.089 |
| ADAM10 | rs442495 | 0.814 | 0.51-1.31 | 0.399 | 0.875 | 0.58-1.32 | 0.526 |
| CLU | rs4236673 | 1.014 | 0.62-1.66 | 0.955 | 0.804 | 0.53-1.22 | 0.301 |
| HLA-DRB1 | rs6931277 | 0.944 | 0.56-1.60 | 0.831 | 0.855 | 0.55-1.34 | 0.496 |

LOAD: late-onset Alzheimer’s disease，SNP: single nucleotide polymorphism, OR: odds ratio, CI: confidence interval

P value was adjusted for gender and age. Bold indicates statistically significant values.
